# Evaluation of Undergraduate Students’ Responsiveness to a 4-Week University-Based Animal-Assisted Stress Prevention Program

**DOI:** 10.3390/ijerph16183331

**Published:** 2019-09-10

**Authors:** Patricia Pendry, Stephanie Kuzara, Nancy R. Gee

**Affiliations:** 1 Department of Human Development, College of Agricultural, Human, and Natural Resource Sciences (CAHNRS), Washington State University, Pullman, WA 99164, USA; 2 Center for Human-Animal Interaction, School of Medicine, Virginia Commonwealth University, Richmond, VA 23298, USA

**Keywords:** human-animal interaction, stress prevention, program evaluation

## Abstract

An increase in the prevalence of stress among college students is compromising their mental health and academic success. One approach to stress prevention that has seen a surge in implementation is the use of university-based Animal Visitation Programs (AVPs). Despite their popularity and promising causal findings, program evaluations on students’ responsiveness (e.g., enjoyment, attendance, perceptions on usefulness and behavioral change) have yet to be conducted. Using a mixed methods approach, this study reports results of a program evaluation embedded in a randomized controlled trial examining the efficacy of incorporating various levels (0%, 50% or 100%) of Human Animal Interaction (HAI) into a four-week long university-based stress prevention program resulting in three conditions: (1) Evidence-based Academic Stress Management content only (0% HAI), (2) Human Animal Interaction with therapy dogs only (100% HAI) and (3) equal combinations of Academic Stress Management and HAI (50% HAI). Responsiveness (e.g., enjoyment, usefulness, recommendation and behavioral change) was assessed quantitatively and qualitatively using self-reported survey data collected immediately following the program and again six weeks later. The results suggest that combining evidence-based content presentations with HAI was associated with higher levels of enjoyment, perceived usefulness, and likelihood of recommendation compared to presenting content presentation or HAI alone, although doing so did not result in differences in perceived behavioral change by condition. Themes of students’ perceptions on the role of HAI in shaping program enjoyment, usefulness, recommendations and behavioral change were described.

## 1. Introduction

There has been a significant increase in university students’ perceived levels of stress [1,2]. According to the American College Health Association’s Executive Summary, 28% of students reported that stress was the most significant factor negatively impacting their individual academic performance, including receiving a lower grade on an exam, course or project, receiving an incomplete, dropping the course, or experiencing a significant disruption in daily academic activities [3] (p. 5). Moreover, although college students’ stress originated from many different sources, academic stress was quoted by 44% of students to be the ‘most traumatic or difficult to handle’ [3] (p. 15). In fact, a great majority of students (84.4%) reported having felt ‘overwhelmed by all they had to do’ at some point during the last 12 months, and 80% reported feeling ‘exhausted, not from physical activity’ [3] (p. 13). The above survey suggests that some university students might experience depressive symptoms. In fact, recent meta-analyses found that 28% of medical students and 34% of nursing students experienced depression [4,5]. Young people with depression may attempt suicide and willingness to seek help is an important protective factor which can reduce suicide risk [6,7]. 

Not surprisingly, campus-based mental health centers report high demand for their services; data from over 93 institutions show that the growth in on-campus counseling center appointments from 2008–2015 (38.4%) was more than seven times the growth in institutional enrollment (5.6%) for that time period [8]. However, according to the Center for Collegiate Mental Health and a study of college student mental health, only 22.3% of students reported ever receiving psychological or mental health services from their current university’s counseling or health services [8,9]. Given the ubiquity of academic stress in university settings, the reluctance of the highest-risk students to seek treatment, and the heavy burden on campus-based mental health centers, it is important to identify and expand evidence-based stress prevention programs to assist students.

### 1.1. Prevalence and Efficacy of University-Based Animal Visitation Programs (AVPs)

One approach to stress prevention that has been enthusiastically received by university administrators and students is Animal Visitation Programs (AVPs). In the U.S. alone, close to a thousand universities offer on-campus AVPs [10]. According to Vandagriff [11], significant variation exists across empirically studied programs regarding aspects such as program duration and type of interaction. Among studies of college-based AAAs, the duration of time in which students could interact with an animal ranged from 7 to 20 minutes. Furthermore, variance existed in the type of interaction, including whether students interacted with animals as a group, the number students per group, or whether interaction occurred one-on-one. The author also noted that the majority of programs involve registered therapy animals that are dogs; however, shelter animals, including cats, have been known to participate in college-based AAAs as well.

Although randomized controlled trials (RCT) are limited, there is promising causal evidence to suggest that brief, university-based AVPs may be effective in reducing students’ perceived stress. For example, an efficacy trial (*N* = 233) examining causal effects of a 10-min long, group-based AVP where students interacted with shelter cats and dogs found significant increases in positive emotion and reductions in negative emotion [12]. Several other causal studies noted beneficial effects on perceived stress [13,14,15], and improvements in mood [16]. Recent findings suggested that effects on mood were not merely perceived; findings suggested that 10 min of hands-on petting of shelter dogs and cats reduced students’ cortisol levels, a marker of the Hypothalamic Pituitary Adrenal (HPA) Axis, one of the body’s stress sensitive systems associated with the development of stress-related disorder [17]. 

### 1.2. Need for Implementation Studies

While efficacy trials examining the effects of university-based AVPs appear to have become more prevalent, program *implementation* evaluations, while important, are rarely conducted. Implementation evaluations are necessary to address the gap between research and practice that has been documented in various fields [18]. In particular, while efficacy trials may demonstrate that an AVP program “does more good than harm when delivered under optimum condition’’, implementation evaluations are necessary to demonstrate AVP effectiveness, meaning that the program “does more good than harm when delivered under real-world conditions” [19] (p. 451). In particular, there is a need to examine an often-neglected aspect of program implementation such as participant responsiveness [20]. Participants’ responsiveness—level of enthusiasm for the intervention—has been operationalized in an integrated program implementation and evaluation model that considers participants’ satisfaction, active participation, home practice, and attendance [20]. Examining responsiveness is important as it is known to be a potential source of disconnect between the program as intended and that which is implemented [21]. Responsiveness has been linked to a number of prevention program outcomes [22,23]. 

Few studies have assessed program participant responsiveness within the field of HAI. A study by Krause-Parello and Morales [24] qualitatively explored the experiences and perceived benefits for veterans with service dogs. Themes that arose included challenges in procuring a service dog, the bond shared between a veteran and their dog, stigma, and psychosocial benefits from having a service dog. However, the relationship between a veteran and their service dog is highly individualized, and research concerning this focused bond may not generalize to more brief and general experiences characteristic of university-based AVPs. Another study assessing the impact of an AAAs on adjudicated female adolescents, participants described what they liked and disliked about the program and reported their feelings about the animals and characteristics of programing [25]. Participants regarded program elements positively, with the majority reporting that they liked petting the animals (91.7%) and learning about the animals (83.3%). However, findings were based exclusively on Likert scale responses rather than extensive thematic qualitative methodology, leaving reflective responses on open-ended questions unexplored.

Thus, using a mixed methods approach, the current study aimed to examine various aspects of participant responsiveness and perceptions of behavioral change in response to participating in a study of a 4-week long university-based animal-assisted stress prevention program that featured various combinations of exposure to HAI and evidenced-based content presentations on academic stress management approaches. A deeper understanding of the components that influence college students’ responsiveness to animal assisted programs may inform program design and implementation, as well as illuminating connections to other implementation dimensions, including efficacy and effectiveness.

## 2. Materials and Methods 

This program implementation evaluation was embedded in an efficacy trial approved by the University’s Institutional Animal Care and Use Committee (ASAF#04785-006), the University’s Institutional Review Board for the protection of human subjects (IRB #14918-005), and the funder’s Animal Welfare and Ethical Review Board (WCR4879). Informed consent was obtained in-person by the PI. Students were told that the purpose of the study was to examine the effects of incorporating HAI into existing stress prevention programs offered at the University. All participants were told they would experience an opportunity to interact with animals at some point during the study, but that the *timing and amount* of HAI would vary by condition. Thus, while students were initially blinded to the expected ratio of exposure to HAI versus content, participants in the condition lacking HAI became aware towards the end of the program that they were deceived about the fact that they would not receive HAI until all outcome assessments and program evaluations had been completed. Students were debriefed about the purpose of this design feature upon study completion and HAI exposure.

### 2.1. Procedure

Student study participation spanned a period of 12 weeks, with the first week spent completing baseline assessments (Week 1), followed by participation in a series of four consecutive weeks of one-hour long programming sessions (Week 2–5), followed by post-test assessments (Week 6), a hiatus of six weeks, and then follow up assessments (Week 12). The study spanned four semesters during which 4 cohorts were recruited using identical procedures. Evaluation surveys were conducted immediately following post-test assessment (Week 6) and after the follow up assessment (Week 12). This manuscript presents results derived from evaluation surveys; Results of the efficacy trial assessments examining effects of condition on various outcomes are reported elsewhere.

### 2.2. Recruitment, Sample and Design

Undergraduate students were recruited in classes in a wide variety of majors, university publications, and by university-based counselors to attend study informational sessions. During informational sessions, students viewed a presentation followed by a question and answer session conducted by the PI. Although designed as a universal prevention program, interested students completed a screening survey that included a masked, randomly assigned condition indicator and questions on several indices of risk associated with stress-related symptoms and academic failure. A general risk indicator was calculated based on participant’s endorsements of at least one of the following items: formerly or presently declared academically deficient, diagnosed with a mental condition or disorder, considered suicide or self-harm, and/or receiving classroom accommodations for learning disorder(s). The risk indicator was used to balance study conditions, and not used as exclusion criteria. We invited participants based on our goal of recruiting a sample with balanced representation by condition of typical and at-risk participants, as well as inclusion of both genders. We continued to extend invitations until we had reached our HAI capacity, which was based on four students per handler-dog team and a maximum of seven handler-dog teams per session. Efficacy trial participants (*N* = 307) were primarily white (*N* = 153, 67.4%), female (*N* = 190), undergraduates (*N*_freshman_ = 118, *N*_sophomore_ = 55, *N*_junior_ = 36, *N*_senior_ = 14, *N*_unknown_ = 5), *M_age_* = 19.11 years (SD = 1.95), and enrolled in an average of 15.3 credits (*SD* = 1.90) with an average GPA of 3.26 (*SD* = 0.66). 

Participants included in the current analyses completed a baseline assessment, attended at least one program session out of four possible program sessions, and completed at least one of the two evaluation feedback surveys conducted after posttest and six weeks later after follow-up assessments (*N* = 228, *n*ASM = 76, *n*HAI-E = 84, *n*HAI-O = 68). Based on a one-way ANOVA to test for between-group differences on all components of the screening survey, we found no significant differences by condition for age (*F*(2, 224) = 1.492, *p* = 0.227, η^2^ = 0.013), sex (*F*(2, 224) = 0.127, *p* = 0.881, η^2^ = 0.001), class standing (*F*(2, 224) = 0.318, *p* = 0.728, η^2^ = 0.003), race (*F*(2, 224) = 1.118, *p* = 0.329, η^2^ = 0.010), risk status (*F*(2, 224) = 2.497, *p* = 0.085, η^2^ = 0.022), GPA (*F*(2, 156) = 0.103, *p* = 0.902, η^2^ = 0.001), or total credits (*F*(2, 221) = 0.340, *p* = 0.712, η^2^ = 0.003).

### 2.3. Conditions

Participants were randomly assigned to one of three conditions at the time of recruitment featuring varying levels of exposure to evidence-based stress management *content* and/or exposure to human animal interaction (HAI). Students randomly assigned to the *Academic Stress Management condition* (ASM) engaged in an existing, evidence-based program using content presentations (e.g., slide presentations featuring evidenced-based information by a health educator) and guided activities focused on enhancing self-regulation (e.g., progressive muscle relaxation, deep breathing, meditation, replacing negative self-talk with positive self-talk) and metacognitive skill training (e.g., time management, test taking skills, study planning, prioritization exercises). Since the featured content was regularly offered as part of a workshop series at the university, this comparison condition was conceptualized as a treatment as usual condition, which did not feature any exposure to animal assisted activities. 

The *Human Animal Interaction condition* (HAI-O) featured semi-structured HAI sessions during which students engaged in guided animal assisted activities (e.g., petting, relaxation activities) with therapy dogs and their handlers for the entire program period without any exposure to evidence-based stress management content. 

Students assigned to the *Enhanced Human Animal Interaction condition* (HAI-E) divided their time equally between engaging in a modified stress management curriculum using the same evidence-based content and activities as described above in the ASM condition and exposure to the same animal assisted activities used in the HAI-O condition where students interacted with therapy dogs and their handlers. 

Program sessions were conducted at prescribed days and times separately from the other conditions to prevent spillover effects or treatment diffusion. As mentioned previously, to avoid condition-specific attrition, all participants, including those in the ASM condition, were told they would experience an opportunity to interact with animals, but that the *timing and amount* of HAI would vary by condition, as such blinding them to the expected ratio of exposure to HAI versus content. HAI was provided to the ASM group after all outcome assessments and program evaluations had been completed. All participants were compensated up to $60 USD for completing assessments, which were prorated at $20 USD per assessment.

### 2.4. Human Animal Interaction: Handler-Dog Teams

Participants in the HAI conditions interacted with registered therapy handler-dog teams who were evaluated members of a regional community partner of the Pet Partners national organization [26]. The teams consisted of 16 male dogs (15 neutered, 1 intact) and 11 female dogs (8 spayed; 3 intact) (*M*_age_ = 4 years, Age_Max_ = 12 years, Age_Min_ = 6 months). The majority of the dogs were Labrador Retrievers (*N* = 10), mixed breeds (*N* = 6), and Golden Retrievers (*N* = 3) (*n_other_* = 8). On average, dogs had been registered with Pet Partners for 1.95 years upon study commencement (range: 1 month–6 years) and participated in 3.6 hours of therapy work per week (range: 1 hour–15 hours per week; includes time spent in the current study and time spent volunteering outside the study). On average, teams participated in 4 program sessions across the four semesters (*session*_min_ = 1, *session*_max_ = 15, *SD* = 3 sessions). The majority of handlers were female (*N* = 24; M_age_ = 49.67; Min = 26, Max = 70), with 2.34 years of AAA experience (range: 1 month–6 years). Each handler-dog team attended an orientation meeting prior to participating, in which the study procedures were explained in detail. During each HAI session, a graduate research student with a degree in animal behavior monitored animal behavior for signs of stress exhibited by the dogs. Handlers were also monitoring their animals’ behavior and wellbeing according to Pet Partner protocols and could freely leave the room if they became concerned about their dog’s well-being.

### 2.5. Description of Setting, Session Outlines, Activities and Themes

All program sessions, including those not featuring HAI, occurred in the same carpeted conference room in a building located at the center of campus, which featured a large center table and perimeter sofas, chairs and side-tables arranged to form seven segmented sitting areas. Each weekly session featured a central theme related to promoting academic success including academic stress management, motivation and goal setting, benefits of sleep, and test anxiety. Specific activities associated with these themes and variations by condition are described in Section 2.5.1, Section 2.5.2, Section 2.5.3 and Section 2.5.4. Students arrived between 5–30 min before program sessions started and were checked in at a front table by the PI and/or graduate students. Students then waited out of view of animals for permission to enter the room which occurred simultaneously. During HAI sessions, each handler-dog team was assigned to one of seven segmented sitting area. Upon entry, groups of 4–5 students were encouraged to approach a handler-dog team of their choosing and to do so while minimizing crowding of the animals. Participants in the ASM condition were encouraged to seat themselves at the center table. 

Regardless of the theme featured that week, program activities were sequenced in the following manner. Students spent the first 20 min of the program in various combinations of receiving evidenced-based content through slide presentations by a Masters level mental health and promotion specialist and/or meeting and/or greeting their peers (ASM) or handler-dog teams (HAI-E/HAI-O). Students assigned to the ASM group received 20 min of content presentations only, those in the HAI-E condition received a combination of 10 min of meet and greet focused HAI and 10 min of content presentations. Participants in the HAI-O group did not receive any evidenced-based content presentations but engaged in 20 min of meet and greet focused HAI. For the remainder of the session, participants engaged in two 10-min-long guided activities; one focused on mindfulness, meditation, relaxation or visualization, and another for small group, semi-structured discussions and reflections. The order of the activities alternated weekly and depending on students’ assigned treatment condition, the facilitation scripts were modified slightly. For participants in groups receiving content (ASM and HAI-E), scripts referred to terminology reflecting information shared during content presentations (i.e., “think about a process-oriented goal”), whereas terminology for the HAI-O group was modified to reflect more general speech (i.e., “think about a reasonable goal”). Additionally, for students assigned to the HAI conditions, guided activities included explicit instructions to touch and stroke the dogs throughout the guided activities. At the end of each session, students in each condition engaged in discussion with peers. HAI-E and HAI-O conditions did so in the presence of the dogs. 

#### 2.5.1. Session 1: Academic Stress Management

Content presentations for students in the ASM and HAI-E focused on manifestations of stress and effective self-care practices to manage stress. Next, participants in each condition were guided through a breathing and body scan exercise. For HAI conditions, this exercise was conducted while sitting and petting and touching the dogs and receiving instruction on ‘experiencing’ the dog they were with. Last, each condition engaged in a discussion activity during which participants sat in small groups with their peers and/or handler-dog teams (HAI-E; HAI-O) in the segmented sitting areas. The discussion activity in the ASM and HAI-E conditions was semi-structured, guided by prompts using terms introduced during the content presentations, focused on identifying and reframing current stressors, as well as discussing students’ use of coping strategies. The HAI-O group engaged in a similar but less structured discussion, using general prompts focused on how animals may help us manage stress. Throughout the discussion activity, participants in the HAI conditions were encouraged to pet and interact with the handler-dog teams as they pleased.

#### 2.5.2. Session 2: Motivation and Goal Setting

Content presentation for students in the ASM and HAI-E focused on identifying and setting attainable goals, establishing behavioral habits to support their completion, including enhancing a growth rather than fixed-mindset, and engaging in self-talk towards goal completion. Next, participants in each condition were guided through a discussion activity during which participants sat in small groups with their peers and/or handler-dog teams (HAI-E; HAI-O) in the segmented sitting areas. The discussion activity in the ASM and HAI-E conditions was semi-structured, guided by prompts using terms introduced during the content presentations, focused on setting attainable academic goals, addressing the anticipated steps necessary, and identifying behavioral modifications towards goals completion. The HAI-O group engaged in a similar but less structured discussion, using general prompts focused on identifying a reasonable academic goal for the semester, why that goal was meaningful, and what barriers they may encounter towards successful completion. Throughout the discussion, participants in the HAI conditions were encouraged to pet and interact with the handler-dog teams as they pleased. Lastly, participants in each condition completed a visualization exercise, during which they were encouraged to witness themselves going through the steps they explored during discussion, concluding with successful completion. For HAI conditions, this exercise was conducted while sitting and petting the dogs. 

#### 2.5.3. Session 3: Benefits of Sleep

Students in the ASM and HAI-E groups received information on the amount of healthy sleep needed, effects of sleep deprivation, and how to overcome common barriers through instruction on identifying behavioral modifications focused on creating optimal sleep environments, routines and behavior. Next, all participants were guided through a progressive muscle relaxation meditation to provide practice at deliberate relaxation to be used as part of a bed-time routine. For HAI conditions, this exercise was conducted while sitting with and touching the dogs. Next, participants in each condition were guided through a discussion activity during which participants sat in small groups with their peers and/or handler-dog teams (HAI-E; HAI-O) in the segmented sitting areas. A similar, semi-structured discussion format using similar prompts was used for each condition focused on exploring the quality of students’ current sleep environments and actions they would be willing to take to improve their sleep environment. For HAI conditions, this exercise was conducted while sitting and petting the dogs. 

#### 2.5.4. Session 4: Test Anxiety

Students in the ASM and HAI-E conditions received information about what test anxiety is, how it can manifest physically and mentally and approaches to overcome anxiety. Next, using identical prompts for each condition, all participants engaged in a visualization activity. For students in ASM and HAI-E groups, this visualization was conducted around the central table, while students in the HAI-O group sat in segmented areas with the dogs. The first 5 min of the activity was intended to evoke feelings of stress and anxiety about a fictional but upcoming exam. The next 10 min consisted of a stress release meditation which included techniques to interrupt disruptive thoughts and feelings, encourage a calm state, and visualize successfully completing the fictional exam. For groups in the HAI conditions, the stress release activity was conducted in the presence of animals and prompted students to think about the dog’s calming presence. Lastly, all students engaged in a discussion activity which focused on reflecting on their experience with the prior activity and how they could utilize the skills practiced in the various mindfulness activities throughout the four program weeks to manage and/or interrupt experiences of stress and anxiety.

### 2.6. Program Fidelity

The program sessions were highly structured with strict adherence to lecture notes by trained facilitation staff and an experienced health educator who facilitated consistently across conditions and throughout each implementation session. The curriculum content was presented with the aid of detailed, memorized scripts. Facilitators adhered to sequences of 30-s intervals which were monitored and prompted by a research assistant who kept a record to document the fidelity of presented content and facilitation of activities. All sessions featuring HAI were video-recorded via seven different simultaneous camera angles.

### 2.7. Measures

#### 2.7.1. Participant Responsiveness

Participants were asked to complete an end of the program evaluation at post-test (Week 6) and again six weeks later, and again after follow-up assessments were completed (Week 12). Although participant surveys were identified by a condition indicator, surveys did not include participants’ ID number to facilitate anonymity of responses. Guided by the integrated theoretical model of program implementation [21], the *satisfaction* aspect of participant responsiveness was assessed at posttest by inquiring about participants’ perceptions of program enjoyment, usefulness, and likelihood of recommending the program to others. Using a 5-point Likert scale, questions assessed the extent to which students *enjoyed* each of four weekly sessions (e.g., 1 = “Not at all enjoyable,” 2 = “Somewhat not enjoyable, “3 = “Neutral,”4 = “Somewhat enjoyable,”5 = Very enjoyable”) and the extent to which students perceived each session as *useful* (e.g., 1 = “Not at all useful,” 5 = “Very useful”). A composite score was calculated for each construct by averaging ratings across the 4-week period, resulting in composite scores for *enjoyment* and *usefulness*. Participants also responded to one question asking how likely they were to *recommend* the program on a 5-point Likert scale (1 = Very unlikely, 5 = Very likely). We also proposed ideas for future program components and asked participants to indicate their preferences about the amount of evidence-based content versus exposure to animal interaction, as well as the extent to which they would like to interact with various animals other than dogs *(0 = not at all interested to 3 = very much interested*) in the context of a future workshop setting on campus. Although the current study focused on examining participants responsiveness, the program evaluation and implementation survey was designed to also evaluate several other common aspects assessed during program implementation (participants’ impressions about facilitation staff, study personnel, engagement with fellow participants etc.) as well as attitudes about animals and animals assisted programs, which were not examined in this study. Participant *attendance* was tracked throughout the 4-week program. 

#### 2.7.2. Perception of Behavioral Change

To capture the *engagement* and *home-practice* component of students’ responsiveness, we assessed the extent to which participants reported changes in behavior across various dimensions and the extent to which they practiced skills at home [21]. We then asked participants to report on the extent to which they used each of these strategies. At post-test, using a 5-point Likert scale, participants rated to what extent participation in sessions improved or did not improve their management of academic stress, motivation, goal setting, sleep habits, and management of test anxiety. A composite score was calculated by averaging ratings across the four themes resulting in a score for *perceived behavioral change*. 

At follow-up, participants completed a feedback survey to assess their perception of change six weeks after program completion. We developed the survey items capturing self-reported perceived behavioral change by listing all the evidenced-based information topics and recommended behavioral strategies for the stress management session, along with that week’s activity and discussion prompts by condition. Students were asked to rate how frequently they engaged in those strategies as a result of programming on a 4-point Likert scale (e.g., 0 = “Not at all,” 3 = “Very Frequently”). These questions were designed to assess the longevity of perceived behavioral change and included 10–14 strategy items for each session theme. Examples of the stress management session strategies included “Recognized when I am feeling stress”, “Positively reframed stress or stressful situations”, “Used progressive muscle relaxation”, “Used meditation or a mental body scan”, “Interacted with animals”, “Engaged in physical activity”, “Ate healthy/healthier”, “Other self-care (saying no, fix small annoyances, etc.)”, “Other, please specify”). In addition to examining perceived behavioral change by condition for each strategy, the average frequency by which students engaged in the above strategies was calculated by averaging rating scores.

### 2.8. Qualitative Data

To better understand the influence of incorporating HAI into program sessions on participant responsiveness and perceptions of behavioral change, participants in the conditions featuring HAI (HAI-E and HAI-O) were asked two open-ended questions to qualitatively describe their favorite and least favorite aspect of interacting with the dogs, i.e., What was your favorite part/least favorite part about interacting with the dogs during program sessions? They were also asked to provide program recommendations (i.e., “Is there anything you recommend we change about content of the programs? Please specify”). Students were also asked to qualitatively describe what they had changed as a result of programming to manage their stress (i.e., “What specific things have you changed in the last 4 weeks to manage stress?”). 

### 2.9. Analytic Strategy

Quantitative data were assessed for normality by examining skewness and kurtosis and conducting Shapiro–Wilk tests. If the Shapiro–Wilk test suggested nonnormality, subsequent nonparametric Kruskal–Wallis H tests were used to determine if constructs representing responsiveness varied across program conditions. If a significant difference was detected, Dunn’s post hoc test with a Bonferroni correction was used to determine how ratings for each item differed [20]. Effect sizes were calculated following Tomczak and Tomczak’s [27] guidelines for calculating the eta-squared measure, which were subsequently converted to Cohen’s *d* [28] for interpretability. 

Qualitative data collected in response to open-ended questions was analyzed by hand by the PI and a doctoral student using thematic analysis, which is a process used to identify and analyze patterns and themes within data [29]. Thematic analysis is a flexible method that is not tied to a particular theoretical or epistemological perspective, making it a suitable method for exploring participants’ experiences. In particular, realist thematic analysis was used, in which focus is placed on reporting the “experiences, meaning and realities of participants” [30]. Analysis followed Braun and Clarke’s six phase framework for doing a thematic analysis, starting first by becoming familiar with the data (1), which allowed the PI and a doctoral student to recognize concepts that appeared frequently and/or consistently. By becoming familiar with the data, a list of initial codes was generated that captured common responses or relevant constructs (2). Responses were recoded and grouped according to these codes in a continuing refinement of codes. This review process continued until the point of saturation was reached, in which no new codes were detected. Codes were then grouped into larger themes that appeared to explain a patterned response in the data (3). Data were then recoded based on these themes and the frequency of codes and themes were calculated and/or ordered by rank. Participant responses that represented those themes well were extracted as examples. Qualitative survey responses were coded according to the emerged coding scheme by another research assistant to determine reliability. In the fourth stage, researchers reviewed and modified themes to tighten the coding process and ensure that themes were accurately capturing what was present in the data (4). The final two steps include defining themes (5) and identifying overarching relationships between themes and (6) writing up results, which are presented below. Thematic analysis can be used to review and analyze qualitative survey responses overall as well compare responses by condition.

## 3. Results

### 3.1. Participant Responsiveness

#### 3.1.1. Enjoyment

The Shapiro–Wilk test suggested non-normality for ratings of enjoyment (*SW* = 0.937, *df* = 218, *p* < 0.001). Thus, nonparametric Kruskal–Wallis H tests were used to determine if participant ratings varied across program conditions. The results showed that participants rated program sessions as somewhat enjoyable (*M*_overall_ = 4.14, *M*_ASM_ = 3.97, *M*_HAI-E_ = 4.24, *M*_HAI-O_ = 4.17) with statistically significant differences across conditions, *Χ*^2^(2) = 7.149, *p* = 0.028, *d* = 0.314, η^2^ = 0.024. A Dunn’s post-hoc test with a Bonferroni correction revealed that participants in the HAI-E group reported significantly higher levels of enjoyment, (z-statistic = −26.938, *p* = 0.024), compared to participants in the ASM-only group, although no significant differences were observed between the HAI-E and HAI-only groups (z-statistic = 9.641, *p* = 1.000) or between the HAI-only and ASM groups (z-statistic = −17.297, *p* = 0.320). Thus, a higher level of enjoyment was reported when participants were exposed to a combination of HAI and stress prevention content compared to engaging only with stress prevention content through lectures and activities. 

Qualitative responses concerning participants identification of their most and least favorite part of interacting with dogs by condition revealed responses across three major themes relating to: (1) the type of HAI *activity* (2) dog *qualities/characteristics* and (3) effects on participants’ *mood*. 

Theme 1: Based on rank ordering, we found that the activity participants in both HAI groups referred to most was *petting* the dogs, followed by being in the dogs’ *presence or proximity* and *mediation activity*. Theme 2: Participants who referred to the dog characteristics as their most favorite part of HAI were most likely to describe the *qualities* that reflected aspects of the animal’s ‘*attitude*’ during the session, particularly referring to dog behavior reflective of *positive affect*, indicated by the use of words such as most enjoying that the dogs appeared *happy, joyful, excited and/or engaged*. Physical traits were mentioned next, indicating that their favorite part of HAI was that the animals were *soft* and *cute*. Theme 3: Participants who mentioned that the most favorite part of HAI was the positive effect the animals had on their *mood* most frequently commented that interacting with the dogs made them feel *happy, joyful and loved*, followed by feeling *relaxed* and *calm*. Quotes best capturing these phenomena included: “I feel as if the animals were my source of comfort when I was stressed” and “They (the dogs) helped me relax and brought a nice break to the material. They also helped me lower my stress”. Based on a counting the number of times participants referred to words capturing themes described above, participants in the HAI-E condition were twice as likely to report that the dogs improved their mood, whereas those in the HAI-O condition most frequently referred to enjoying that the animals cleared their minds and took their attention away from stress or school. 

Qualitative responses concerning participants’ identification of their least favorite part of interacting with dogs by condition revealed responses that were classified into the same themes including: (1) the type of HAI *activity* (2) dog *qualities/ characteristics* and (3) effects on participants’ *mood*. 

Theme 1: Based on rank ordering, we found that the least favorite part of HAI activities was *closing ones’ eyes* during meditation in the presence of animals. This was experienced as *distracting* or *uncomfortable*, as participants were concerned about inadvertently *touching other people’s hands* or being *unable to track the animals’ movement*. Interestingly, this patterns was common for HAI-E participants whereas HAI-O participants who engaged in the exact same meditation activities as the HAI-E group did not comment on this at all. Theme 2: Participants who referred to the dog characteristics or qualities as their least favorite part of HAI, were most likely to dislike the animals’ *shedding*, which was commented on equally frequently by participants in both conditions. Theme 3: Participants who identified negative experiences of HAI on their mood were most likely to point out experiencing *limited access* to the animals reduced their enjoyment; participants in the HAI-O group most often commented on not liking *having to share* animals with others, while HAI-E participants most often commented on finding *rotations* between dogs challenging. Additionally, HAI-E second most pressing negative influence on enjoyment was feeling challenged by engaging in materials that made them *think about stress*. 

#### 3.1.2. Usefulness

The Shapiro–Wilk test suggested nonnormality for ratings of usefulness (*SW* = 0.956, *df* = 218, *p* < 0.001). On average, participants rated program sessions as somewhat useful (M_overall_ = 3.95, M_ASM_ = 3.84, M_HAI-E_ = 4.11, M_HAI-O_ = 3.87). A Kruskal–Wallis H test showed that there was a statistically significant difference in usefulness across conditions, Χ^2^(2) = 6.729, *p* = 0.035, d = 0.300 η^2^ = 0.022. Dunn’s post-hoc test with a Bonferroni correction revealed that participants in the HAI-E group reported significantly higher levels of usefulness compared to participants in the HAI-only group (z-statistic = −22.540, *p* = 0.031), and significantly higher levels compared to participants in the ASM-only groups (z-statistic = −23.069, *p* = 0.023). Effect size was calculated, suggesting that 2.2% of variability in usefulness scores was accounted for by condition assignment. Cohen’s *d* for usefulness was medium (d = 0.30) according to Cohen [22] criteria. There were no significant differences between the HAI-only and ASM groups (z-statistic = 0.529, *p* = 1.00). Thus, a higher level of usefulness was reported when structured evidence-based workshop material was combined with HAI, compared to only HAI or only content-based material. 

Although a few students in the HAI-E group noted that the animals were *irrelevant* to the material (2.9%) or *distracting* (2.9%), these comments were rare compared to those stating the presence of dogs helped students *focus on the material*. For example, a student commented that: “Having an animal to touch/feel made taking my mind off stress much easier. Shifting focus is easier with an animal”, while another student commented: “I felt that the animals made me feel less stressed, and energized enough to listen to the presentations to the best of my ability”. Several students (14.6%) commented that the dog’s presence made them more motivated to attend and participate during sessions, commenting, “It made the program overall more enjoyable because we had to opportunity to interact with animals”. Other students noted that, “without the dogs, this would have just felt like another class or lecture-boring”, and “it gave me something to look forward to” and “the animals were the reason I came and really, the only thing that made me feel more calm”.

#### 3.1.3. Recommendation

The Shapiro–Wilk test suggested non-normality for ratings of recommendation likeliness (*SW* = 0.677, *df* = 218, *p* < 0.001). On average, participants reported a highly likelihood of recommending the program to others (M_overall_ = 4.28, M_ASM_ = 4.04, M_HAI-E_ = 4.38, M_HAI-O_ = 4.35). A Kruskal–Wallis H test showed that there was a statistically significant difference in likelihood of program recommendation across conditions, Χ^2^(2) = 11.234, *p* = 0.004, d = 0.424, η^2^ = 0.043. Effect size calculations suggest that nearly 4.3% of variability in recommendation scores was accounted for by condition assignment. The effect size for recommendation likeliness was medium (d = 0.424). Dunn’s post-hoc test with a Bonferroni correction revealed that participants in both HAI groups were significantly more likely to recommend the program compared to participants in the ASM-only group (HAI-O versus ASM-only: z-statistic = −26.804, *p* = 0.016; HAI-E versus ASM-only: z-statistic = −27.402, *p* = 0.008), although no significant differences were observed between the HAI-E and HAI-only groups, z-statistic = 0.598, *p* = 1.00. Thus, participants in conditions with animal interaction (HAI-E and HAI-O) reported a greater likelihood of recommending the program compared to the condition in which no animals were present (ASM).

When asked to indicate their preferred balance of HAI and evidence-based content exposure, 85% of students reported a preference for a combination of 75% animal interaction and 25% content information, while only a small proportion of students reported wanting only HAI (14%), and an even smaller percent of students wanted only information and no HAI (1.5%). Finally, to explore student interest in animals other than dogs, we conducted a paired-samples t-test to examine the extent to which participants indicated they liked to interact with animals other than dogs in future campus workshops *(0 = not at all interested to 3 = very much interested*) and found a significant difference by animal type with participants clearly preferring interacting with dogs (*M* = 2.94, *SD* = 0.248) compared to cats (*M* = 2.23, *SD* = 0.110; *t*(61) = 5.188, *p* = 0.0001), rabbits (*M* = 2.13, *SD* = 1.01; *t*(61) = 6.21, *p* = 0.0001), horses (*M* = 1.80, *SD* = 1.17; *t*(61) = 7.53, *p* = 0.0001), guinea pigs (*M* = 1.54, *SD* = 1.18; *t*(61) = 9.16, *p* = 0.0001), and goats (*M* = 1.75, *SD* = 1.21; *t*(61) = 7.58, *p* = 0.0001). Based on a one-way ANOVA conducted to compare the effect of treatment condition on level of interest in dogs, results indicate there were no significant differences by condition (F(2, 59), *p* = 0.894).

Many students contextualized these recommendations by expressing that interacting with animals was *relaxing and enjoyable*, while receiving information was *stressful or redundant*. Some students expressed that receiving information only served to conjure thoughts of stressful situations or realities without providing a means of remedying the source of the stress. For example, one student commented, “I feel like when we think of difficult things or things we’re unable to do because we’re in the room for an hour it creates more stress or doesn’t apply to our head space at the time. Animals help enough with destressing and make me happy”. Another student commented that receiving information felt like more schooling, stating, “When info was given to me I felt like I either already knew it or overwhelmed me more because since school has been so hard I felt like I was doing more work and being lectured even more. Playing with the dogs was much more relaxing and beneficial”. Beyond feeling like just an extended day in the classroom, some students commented that sessions repeated information students had received from other workshops or settings, such as Greek life. These comments provide context for why the majority of students favored animal interaction over information. 

However, students did not find stress management content entirely useless or frustrating. Rather, the majority of students (53%) expressed a *desire for being exposed to a combination* of the two. Some students commented that animals were relaxing in the moment but receiving information about how to manage stress would be useful in the future. For example, one student summarized this sentiment by stating, “Animals can only do so much. I need steps that I can do to help fix the problems I have in my life”, and another student responded, “The animals are calming, but accurate information is important too. People will manage problems better if they can actually understand them”. The perception that the animals helped engage with content resonated with many students stating, “I find the info fascinating and helpful. The animals make it better because they help me relieve my stress so the methods stick better”, while another student commented, “If too much talking, it feels like I’m in class. Animals help the environment seem to be like a playground. Make me forget about stress”. The presence and engagement with animals appeared to serve as a momentary source of relaxation as well as a *motivator for involvement with content*, which may enhance adaptation of student adaptive stress management techniques and coping for long term advantage. 

### 3.2. Attendance

On average, participants attended the majority of the four sessions (*M* = 3.54 sessions, *SD* = 0.69, *Min* = 1, *Max* = 4). A one-way ANOVA showed no statistically significant differences in the overall number of sessions attended by group (*F*(2) = 1.084, *p* = 0.340 (*M*_HAI-O_ = 3.49 sessions, *M*_ASM_ = 3.50 sessions, *M*_HAI-E_ = 3.63 sessions)), reducing the potential threat to internal validity caused by selective attrition. 

### 3.3. Behavioral Change

A Shapiro–Wilk test suggested nonnormality for ratings of behavioral change (*SW* = 0.902, *df* = 218, *p* < 0.001). Thus, nonparametric tests were used to examine whether differences emerged by condition in participants rating of their behavioral change. On average, participants reported some improvement in their management of stress as a result of program participation (*M*_overall_ = 3.58, *M*_ASM_ = 3.49, *M*_HAI-E_ = 3.68, *M*_HAI-O_ = 3.57). A Kruskal–Wallis H test showed that there were no statistically significant differences in *participant-rated perception of change* by condition, *Χ*^2^(2) = 2.351, *p* = 0.309, *d* = 0.090, η^2^ = 0.002. The eta-squared measure (η^2^) effect size suggested that 0.2% of variability in change scores was accounted for by condition and Cohen’s *d* = was small (*d* = 0.090).

#### Home Practice at Follow-Up

**Figure 1:**
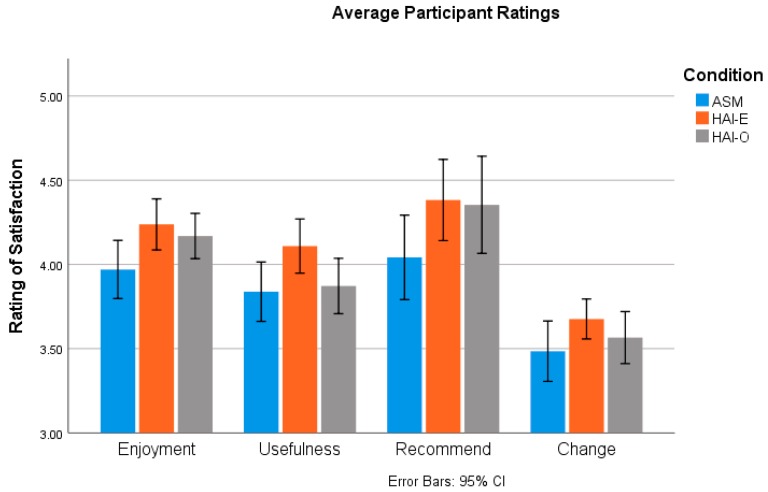
Ratings of responsiveness by condition.

A Shapiro–Wilk test suggested normality for home practice completion (*SW* = 0.988, *df* = 212, *p* = 0.063). Students reported, on average, engaging in program strategies occasionally to manage stress (*M* = 1.38, *SD* = 0.48, *Min* = 0.22, *Max* = 2.78), with no statistical differences in overall ratings of strategy engagement by condition, *F*(2) = 0.833, *p* = 0.436, *d* = 0.180, η^2^ = 0.008. The most commonly used strategy by students was recognizing when they were feeling stressed (*M* = 2.19, *SD* = 0.76, *M*_ASM_ = 2.17, *M*_HAI-E_ = 2.23, *M*_HAI-O_ = 2.18) and engaging in other self-care activities (*M* = 1.78, *SD* = 0.96, *M*_ASM_ = 1.74, *M*_HAI-E_ = 1.91, *M*_HAI-O_ = 1.66). Interestingly, although not ranked as a frequent coping strategy in any group, the HAI-O group relied more on visualizing interacting with animals (M = 1.23) than actually interacting with animals (M = 0.089) after program completion. In contrast, the ASM (M = 1.19) and HAI-E group (M = 1.19) were more likely to endorse actually interacting with animals more and less on visualizing interacting with animals (*M*_ASM_ = 0.86, *M*_HAI-E_ = 1.11). A one-way ANOVA found that these differences were not significant (visualized interacting with animals, *F*(2) = 2.169, *p* = 0.117, *d* = 0.30, *η^2^* = 0.021; interacted with animals, *F*(2) = 1.725, *p* = 0.181, *d* = 0.26, *η^2^* = 0.016). Rating of responsiveness and perceptions of behavioral change are presented and summarized in Figure 1. 

## 4. Discussion

The results from this study represent initial efforts to describe and understand the experiences and perspectives of students engaging in a 4-week long university-based, animal-assisted stress prevention program. Understanding participants’ responsiveness and their perceptions about behavioral change is important for determining the extent to which incorporating HAI enhances program efficacy and effectiveness. 

Students reported all three conditions to be enjoyable and useful, with the majority of students expressing a likelihood of recommending either program to others. Students assigned to the condition with 50% exposure to HAI and 50% exposure to stress-prevention programing (i.e., the HAI-E condition) consistently reported higher levels of enjoyment, perceived usefulness, and likelihood of recommendation than students in either of the other two conditions. This suggests that incorporating HAI with existing stress prevention programming may increase participant enjoyment and likelihood of recommendation which may, in turn, improve student engagement, and the efficacy of the program in reducing stress and improving academic performance. 

Theoretical models suggest that students who enjoy curriculum or programming are more likely to be engaged and more likely to benefit from delivered material [31]. The idea that highly engaged students who enjoy program content will fare better in terms of program outcomes can be applied when assessing the efficacy of a university-based stress prevention program. By incorporating HAI, programs are deemed more enjoyable by students, which in turn increases student engagement and promotes positive program outcomes. Therefore, by considering evidence that greater participant self-reported enjoyment is strongly related to more positive program outcomes [32], results from the current study suggest that incorporating HAI may be beneficial toward increasing programming success. However, it still needs to be determined which program outcomes are influenced by student engagement in the particular context of stress-prevention programming. On the other hand, the results indicate that it is important to consider aspects that may compromise enjoyment. In addition to the negative perceptions about being subject to animal shedding, the experience of limited access through sharing with others or having to rotate between handler-dog teams can detract from positive experiences. It is important that we consider the type of activities we encourage students to engage in as well as the structure we impose. For example, although meditation in the presence of animals was considered enjoyable, participant discomfort with being asked to close their eyes while petting is important to consider when assessing programming. Being unable to track an animal’s behavior extends beyond feelings of discomfort to represent a potential safety issue and limitation in programming. 

The HAI-E condition may have been perceived as more useful than the other two conditions because interacting with a therapy dog may serve as positive/rewarding stimulus that becomes associated with the ASM content. When paired with unpleasant stimuli, such as discussing stressors, the dogs may serve as a positive/rewarding stimulus that buffers the associated stress and signals to humans that the environment is nonthreatening. Thus, the HAI-E condition was perceived to be more useful than the stress prevention program only condition because the presence of animals created a more positive and calming environment that allowed for optimal onboarding of ASM content. However, animals alone were not deemed as useful as a combination of animal interaction and stress prevention material. Students may experience enjoyment and an immediate stress reducing impact of the dog, but they may also recognize that having interacted with the dog alone is unlikely to have a longer-term impact on academic challenges. Therefore, while animals may help to create a relaxing and optimal environment, animal interaction alone is probably not useful for learning stress prevention or coping strategies. These results suggest that the incorporation of HAI into evidence-based stress prevention programming increases participant enjoyment while still being perceived as useful. 

The trend of HAI-E being rated highly continues when assessing the reported level of perceived change. Students in the HAI-E group reported a higher level of improvement in their management of stress as a result of program participation than other groups, although differences were not significant. Given that the primary goal of ASM programming is to deliver a beneficial change to participants, these results are encouraging. Thus, incorporating HAI into evidence-based, stress prevention programming may serve to improve participant responsiveness to such evidence, which eventually may lead to greater efficacy and effectiveness of such programs. That said, program *success* is likely to depend on the needs and characteristics of the populations served, the outcomes targeted and the extent to which implementation fidelity is achieved. 

Future research should examine the dosage of evidence-based, stress prevention material required to promote positive behavioral change and the dosage of HAI required to improve perceived enjoyment and usefulness, while at the same time tracking academic performance to assess applied outcomes. Future research should also consider the stress management strategies that students adopted most frequently and readily. It may be a matter of encouraging techniques that easily fit into a student’s life to insure a greater likelihood of successful adoption and change. Such strategies were extracted from qualitative analyses and included students adopting healthy habits (e.g., better diet, exercise) and reframing stress (e.g., seeing challenges and opportunities instead of road blocks). Students were less likely to use strategies that may be more foreign to them, such as mental body scans or progressive muscle relaxation. The low frequency of these strategies may suggest that students need more time or more direction about how to incorporate these skills into their everyday lives. In addition, it would be useful to examine participants’ perceived behavioral change in response to programming themes beyond stress management such as those aimed at affecting behavior in domains related to motivation and goal setting, sleep behavior and management of test anxiety, which were not analyzed in this paper. Future research should also further unpack the role of gender, student standing and risk-status in informing individual differences in program responsiveness and behavioral change to enhance our ability to design program activities targeted to specific populations. Finally, it is important to recognize that it is not always feasible to prevent stress. Students are inevitably going to experience high degrees of stress, and it may be more useful to teach strategies for coping with stress to avoid reaching a state of being overwhelmed by stress. 

## 5. Conclusions

The results from feedback surveys collected following participation in a university-based stress prevention program incorporating HAI provide insight into the participant experience. Programming that incorporates both interactions with animals and evidence-based stress prevention programming was reported to be more enjoyable and useful, with more students reporting a likelihood of recommending the program to others. 

Future research on university-based AAAs is needed to explore additional program implementation dimensions theorized to influence the delivery of programming. Other dimensions, such as dosage, reach, fidelity, and adaptation, need to be considered to fully evaluate and inform the implementation of AAAs. Overall, this study represents initial efforts to recognize the participant’s experience within university-based AAAs and assess how varied levels of HAI and evidence-based stress prevention programming influence participant responsiveness and perceived behavioral change. Finally, researchers, practitioners and University administrators should continue to consider whether encouraging results from efficacy trials and program evaluations on human functioning and wellbeing warrants the inclusion of animals in stress prevention programs as ensuring their safety and wellbeing is equally important.
